# Real-world adverse event profiles of Zuranolone and Brexanolone based on FAERS and VigiAccess databases: An observational study

**DOI:** 10.1097/MD.0000000000049845

**Published:** 2026-07-17

**Authors:** Guanbo Xie, Huili Weng, Jingxian Yu, Liqun Chi

**Affiliations:** a Department of Pharmacy, HaiDian District Maternal and Child Health Care Hospital, Beijing, China; b Department of Pharmacy, Chengde Maternity and Child Health care Hospital, Chengde, Hebei, China.

**Keywords:** adverse events, brexanolone, FAERS, postpartum depression, VigiAccess, Zuranolone

## Abstract

Zuranolone and Brexanolone are Food and Drug Administration-approved novel agents for postpartum depression, yet real-world post-marketing safety data for the two drugs remain insufficient. This study aimed to systematically characterize their adverse event (AE) profiles via two authoritative pharmacovigilance databases, namely the Food and Drug Administration Adverse Event Reporting System (FAERS) and VigiAccess. FAERS data from Q1 2019 to Q3 2025 and VigiAccess data updated to November 2025 were extracted. Reporting odds ratio and proportional reporting ratio were adopted to screen and compare AE signals of the two drugs. A total of 631 primary suspect (PS) drug reports for Zuranolone and 102 for Brexanolone were extracted from FAERS, whereas 863 Zuranolone and 227 Brexanolone reports were obtained from VigiAccess. At the system organ class level, AEs associated with Zuranolone involved 22 SOCs in FAERS and 24 SOCs in VigiAccess, with the top three SOCs sorted by reported cases as follows: Nervous system disorders, Psychiatric disorders, and General disorders and administration site conditions (FAERS); Nervous system disorders, General disorders and administration site conditions, and Psychiatric disorders (VigiAccess). For Brexanolone, AEs retrieved from FAERS involved 16 SOCs, compared with 21 SOCs in VigiAccess; the top three SOCs by the number of reported cases were Injury, poisoning and procedural complications, General disorders and administration site conditions, and Psychiatric disorders. After signal screening in the FAERS database, a total of 78 positive signals (involving 9 SOCs) were identified for Zuranolone, whereas 10 positive signals (involving 4 SOCs) were detected for Brexanolone. Four SOCs were commonly implicated in the positive AE signals of both drugs, with the most commonly reported AEs including gait disturbance, exposure via breast milk, fatigue, and therapy cessation. Additionally, compared to Brexanolone, 5 SOCs were uniquely associated with Zuranolone, with the most commonly reported AEs such as vertigo, vision blurred, muscle twitching, somnolence, and loss of personal independence in daily activities. Zuranolone’s AEs mainly involved nervous system disorders and Psychiatric disorders, while Brexanolone’s primarily included Injury, poisoning and procedural complications, and Psychiatric disorders. This study would provide more safety reference data for the clinical use of two drugs.

## 1. Introduction

Postpartum depression (PPD) represents one of the most prevalent mental health conditions among women in the postpartum period, characterized by prominent depressive mood, persistent anxiety, irritability, intense feelings of isolation, and even suicidal ideation or thoughts of infant-directed harm.^[1,2]^ This disorder exerts profound adverse impacts not only on the maternal physical and psychological health and family dynamics but also on the long-term growth and development of offspring, with potential consequences including low birth weight, developmental delays, and the onset of emotional and behavioral disturbances during childhood.^[3–5]^

Current pharmacological interventions for PPD primarily encompass selective serotonin reuptake inhibitors (SSRIs), serotonin-norepinephrine reuptake inhibitors, and tricyclic antidepressants. Of these agents, SSRIs are generally regarded as the first-line therapeutic option for PPD; nonetheless, a growing body of research has identified several notable limitations, including a delayed onset of therapeutic effects and suboptimal clinical efficacy in certain patient populations.^[6–9]^ As the first drug explicitly approved for the treatment of PPD, Brexanolone was granted authorization by the U.S. Food and Drug Administration (FDA) in 2019.^[10]^ In 2023, Zuranolone became the first oral medication to secure FDA approval for the treatment of PPD, and it shares the same pharmacological target as Brexanolone.^[11]^ Functioning as a positive allosteric modulator (PAM), the two agents exert its pharmacological effects by selectively binding to γ-aminobutyric acid type A (GABA_A_) receptors. Unlike traditional antidepressants that rely on monoamine neurotransmitter regulation, Zuranolone and Brexanolone exerts its therapeutic effects by enhancing the amplitude and duration of GABA_A_ receptor-mediated inhibitory postsynaptic potentials, thereby fine-tuning GABAergic neurotransmission and rapidly reestablishing the excitation-inhibition equilibrium within the central nervous system (CNS).^[11,12]^

Zuranolone and Brexanolone are both PAMs of the GABA_A_ receptor for PPD, yet they differ substantially in administration routes, launch timelines, and clinical monitoring requirements. Brexanolone is delivered via 60 hours of continuous intravenous infusion and requires mandatory in-hospital real-time monitoring throughout treatment, largely to guard against excessive sedation and abrupt loss of consciousness.^[12,13]^ In contrast, zuranolone is an oral formulation taken once nightly at 30 to 50 mg over a 14-day treatment course.^[14]^ Multiple Phase III clinical trials have verified its rapid onset of action and sustained antidepressant effects up to day 45.^[15,16]^ Nevertheless, the available safety evidence for both agents mainly originates from short-term controlled clinical trials, which are restricted by limited sample sizes, short follow-up periods, and strict eligibility criteria.^[17]^ Few head-to-head post-marketing pharmacovigilance studies have systematically compared their adverse event (AE) profiles in routine clinical practice. Large-scale post-marketing spontaneous reporting databases can compensate for these inherent limitations of clinical trial data. The U.S. FDA Adverse Event Reporting System (FAERS) contains a wealth of real-world AE data pertaining to pharmaceuticals marketed in the U. S.^[18,19]^ while the World Health Organization - managed VigiAccess database aggregates global AEs for pharmacovigilance research worldwide.^[20,21]^ Accordingly, a head-to-head safety signal analysis of Brexanolone and Zuranolone was conducted using these two databases. Comparing their safety profiles can identify the limitations of early PPD neuroactive steroid therapies, provides valuable historical reference for optimizing the development of subsequent novel neuroactive steroids.

## 2. Methods

### 2.1. Data source

**Figure 1.**
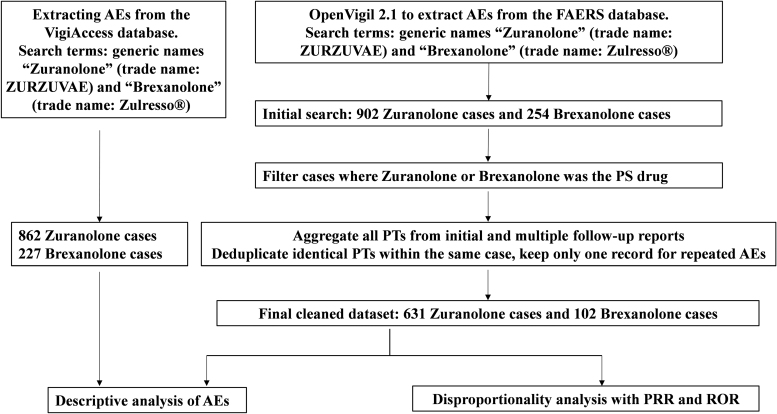
Flow diagram of this study. AEs = adverse events, FAERS = FDA adverse event reporting system, PS = primary suspect, PT = preferred term, PRR = proportional reporting ratio, ROR = reporting odds ratio.

The data utilized in this study were derived from anonymized AE reports retrieved from two publicly accessible pharmacovigilance databases: FAERS and VigiAccess. OpenVigil 2.1 (https://openvigil.sourceforge.net/), a well-validated and widely cited pharmacovigilance analysis platform, was employed to extract, clean and mine FAERS-related data.^[22,23]^ Specifically, the generic names “Zuranolone” (trade name: ZURZUVAE) and “Brexanolone” (trade name: Zulresso®) were used as search terms to identify AE reports where each drug was classified as the primary suspect (PS) drug. The FAERS data extraction spanned the period from the first quarter of 2019 (Q1 2019) to the third quarter of 2025 (Q3 2025). Concurrently, the VigiAccess database was queried using the same generic drug names, with data retrieval updated through November 2025. The flowchart of this study is shown in Figure 1.

### 2.2. Data processing and standardization

AE information was standardized and categorized using the Medical Dictionary for Regulatory Activities (version 27.0). Each AE record was assigned a preferred term (PT) and further classified into different systems according to the system organ class (SOC).

### 2.3. Statistical analysis

Disproportionality analysis serves as a core methodology in pharmacovigilance research, enabling the detection of potential medication-associated AEs by comparing the incidence of specific AEs between a target drug and all other drugs within a dataset, based on a 2 × 2 contingency table framework (Table 1).^[20]^ The reporting odds ratio (ROR) method and the proportional reporting ratio (PRR) method in the disproportionality analysis were employed to identify risk signals from the AE reports extracted from the FAERS database.^[18,20]^ The mathematical formulas for calculating PRR and ROR are detailed in Table 2. Signal detection followed predefined thresholds to ensure statistical rigor: a minimum of 3 AE reports (denoted as “a” in Table 1) was required for initial consideration. A positive signal was further confirmed if the lower bound of the two-tailed 95% confidence interval (95%) for ROR exceeded 1 and PRR ≥ 2 and the chi-squared (χ^2^) ≥ 4. All statistical analyses were performed using Microsoft Office Excel 2019 and R software (version 4.5.2; R Foundation for Statistical Computing, Vienna, Austria).

**Table 1:** 2 × 2 contingency table for disproportionality analysis.

Project	Drug(s) of interest	All other drugs	Total
Adverse event (s) of interest	a	b	a + b
All other adverse events	c	d	c + d
Total	a + c	b + d	a + b + c + d

**Table 2:** Calculation formulas and thresholds for ROR and PRR.

Algorithms	Equation	Criteria
ROR	ROR = ad/bc 95%CI = e^lnROR±1.96(1/a+1/b+1/c+1/d)0.5^	a ≥ 3, lower limit of 95% CI > 1
PRR	PRR = a(c + d)/c(a + b) χ^2^ = ([ad-bc]^2^)(a + b + c + d)/([a + b][c + d][a + c][b + d])	a ≥ 3, PRR ≥ 2, χ^2^ ≥ 4

CI = confidence interval, PRR = proportional reporting ratio, ROR = reporting odds ratio.

### 2.4. Ethical considerations

This study was based on publicly available, de-identified data from FAERS and VigiAccess databases. Therefore, ethical approval and informed consent were not needed.

## 3. Results

### 3.1. Descriptive analysis

From Q1 2019 to Q3 2025 in the FAERS database, a total of 631 reports were identified where Zuranolone was listed as the PS drug. For Brexanolone, 102 reports were retrieved from FAERS during the same period as the PS drug. As of November 2025, 863 Zuranolone-related reports and 227 Brexanolone-related reports as the PS drug were extracted from the VigiAccess database.

In addition to cases with missing demographic data, male patients were documented among users of both drugs in both databases. Regarding age distribution, patients aged 18–45 years accounted for the largest proportion in both drug groups. Geographically, all AE reports of the two drugs in both databases were originated from the U. S. Temporally, the number of Zuranolone-related AE reports showed a sustained upward trend over the reporting period. In contrast, Brexanolone-related AE reports were concentrated in 2022–2024 (FAERS) and 2020–2023 (VigiAccess). Serious outcomes associated with Zuranolone included death, hospitalization, life-threatening conditions, and other medically important events, while those linked to Brexanolone were mainly hospitalization and other medically important events. Detailed clinical characteristics of Zuranolone- and Brexanolone-related cases are summarized in Table 3 (FAERS) and Table 4 (VigiAccess).

**Table 3:** Clinical characteristics of Zuranolone- and Brexanolone-associated AE reports from the FAERS database (Q1 2019–Q3 2025).

Characteristics	Subgroups	Zuranolone		Brexanolone	
		Case number, n	Case proportion, %	Case number, n	Case proportion, %
Number of events		631		102	
Gender	Female	573	90.81	76	74.51
	Male	2	0.32	4	3.92
	Unknown	56	8.87	22	21.57
Age	<18 yr	0	0	6	5.88
	18–35 yr	226	35.82	43	42.16
	36–45 yr	72	11.41	10	9.80
	Unknown	333	52.77	43	42.16
Reported Countries	US	631	100	102	100
Yr	2019	–	–	4	3.92
	2020	–	–	4	3.92
	2021	–	–	4	3.92
	2022	–	–	32	31.37
	2023	1	0.16	36	35.30
	2024	361	57.21	21	20.59
	2025	269	42.63	1	0.98
Serious Outcomes	Death	1	0.16	0	0
	Hospitalization - initial or prolonged	17	2.69	1	0.98
	Life-threatening	2	0.32	0	0
	Other serious (important medical event)	62	9.83	2	1.96
	Required intervention to prevent permanent Impairment/damage	3	0.47	0	0
	Unknown/blank	546	86.53	99	97.06

**Table 4:** Clinical characteristics of Zuranolone- and Brexanolone-associated AE reports from the VigiAccess database (up to November 2025).

Characteristics	Subgroups	Zuranolone		Brexanolone	
		Case number, n	Case proportion, %	Case number, n	Case proportion, %
Number of events		863		227	
Gender	Female	774	89.69	140	61.68
	Male	3	0.35	6	2.64
	Unknown	86	9.96	81	35.68
Age	<18 yr	1	0.12	13	5.73
	18–44 yr	397	46.00	98	43.17
	45–64 yr	3	0.35	0	0
	Unkown	462	53.53	116	51.10
Reported Countries	US	863	100	227	100
Yr	2019	–		6	2.64
	2020	–		56	24.67
	2021	–		41	18.06
	2022	–		47	20.71
	2023	0	0	42	18.50
	2024	255	29.55	23	10.13
	2025	608	70.45	12	5.29

Further, serious outcome reports in FAERS where Zuranolone and Brexanolone were designated as the PS drug were summarized (Supplementary Table 1, Supplemental Digital Content 1). For Zuranolone, the death category included 2 PTs including completed suicide; the initial or prolonged hospitalization group had 48 PTs with suicidality-related items of suicidal ideation, suicide attempt and intentional overdose. Suicidal ideation existed in the 5 PTs of life-threatening events, while the other medically important events (24 PTs) contained suicidal ideation, self-injurious ideation and homicidal ideation. No suicidality-related AEs were found in the 13 PTs requiring intervention to prevent permanent impairment. For Brexanolone, only 7 PTs for hospitalization and 4 PTs for other medically important events were retrieved, with no suicidality-related AEs in either group.

### 3.2. SOC distribution of AEs reported for Zuranolone and Brexanolone in two databases

In the FAERS database, based on 631 PS drug reports, a total of 1666 AEs associated with Zuranolone were reported, involving 22 SOCs. Among these, Nervous system disorders ranked first (562 cases, 33.73%), followed by Psychiatric disorders (537 cases, 32.23%) and General disorders and administration site conditions (266 cases, 15.97%). For Brexanolone, based on 102 PS drug reports, 194 AEs were documented in the FAERS database, covering 16 SOCs; here, Injury, poisoning and procedural complications were the most prevalent SOC (65 cases, 33.51%), succeeded by General disorders and administration site conditions (44 cases, 22.68%) and Psychiatric disorders (34 cases, 17.53%).

Consistent trends were observed in the VigiAccess database, where 1629 Zuranolone-related AEs (involving 24 SOCs) were reported. The top three SOCs for Zuranolone were similar to those in FAERS: Nervous system disorders (590 cases, 36.22%), General disorders and administration site conditions (332 cases, 20.38%), and Psychiatric disorders (322 cases, 19.77%). For Brexanolone, 382 AEs (involving 21 SOCs) were recorded in VigiAccess, with a top-three SOC distribution identical to that in FAERS: Injury, poisoning and procedural complications (108 cases, 28.27%) ranked first, followed by General disorders and administration site conditions (88 cases, 23.04%) and Psychiatric disorders (64 cases, 16.75%).

**Figure 2.**
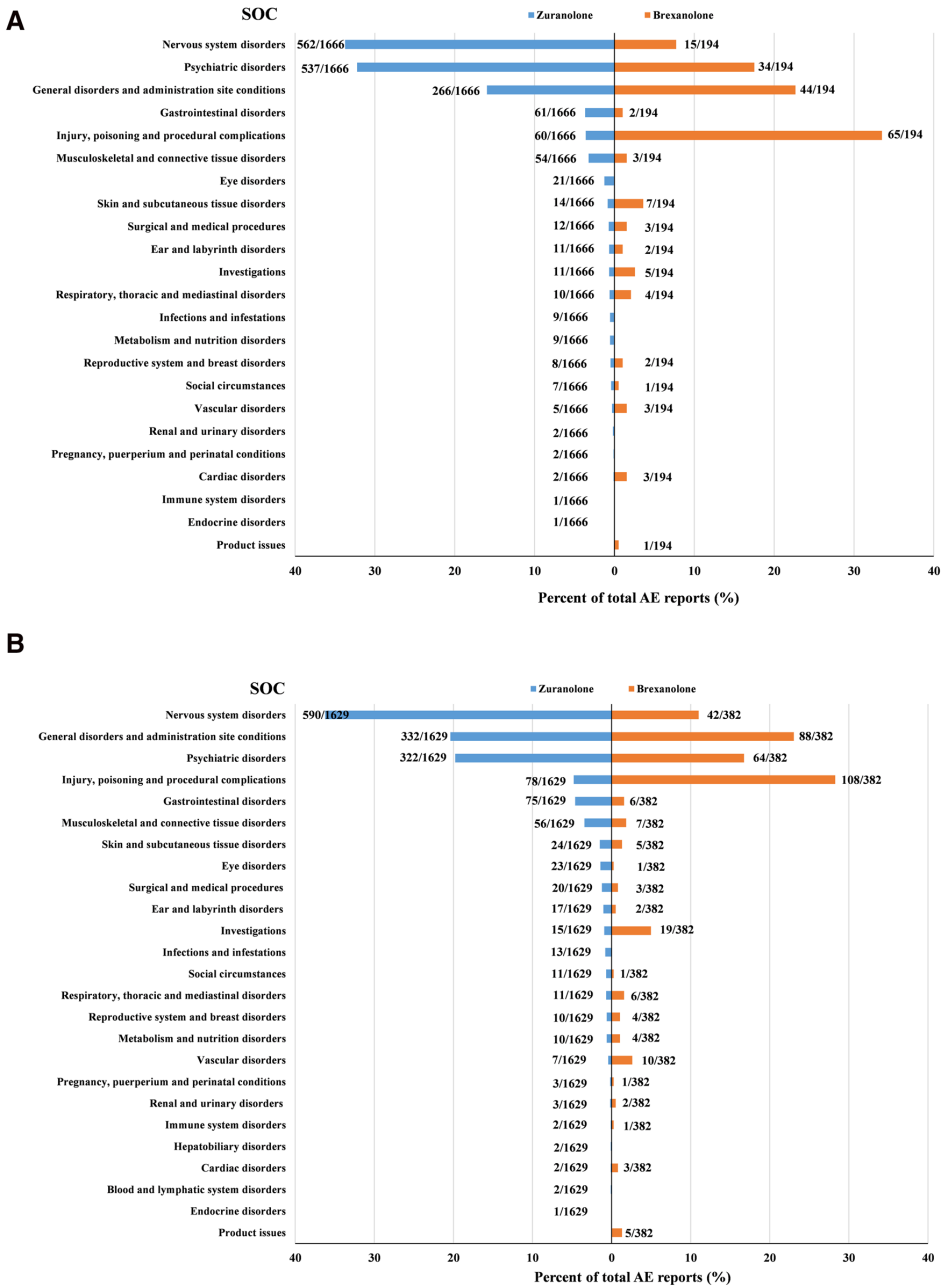
Distribution of SOC for Zuranolone and Brexanolone in FAERS database (A) and VigiAccess database (B); Numerical labels on bars denote (AE reports of individual SOC)/ (total AE reports of each drug). SOC = system organ class.

Notably, the SOCs involved in AEs of both drugs were nearly identical across the two databases. Detailed distributions of AEs-related SOCs for the two drugs are presented in Figures 2A and 2B.

### 3.3. Signal distribution at the PT level

**Figure 3.**
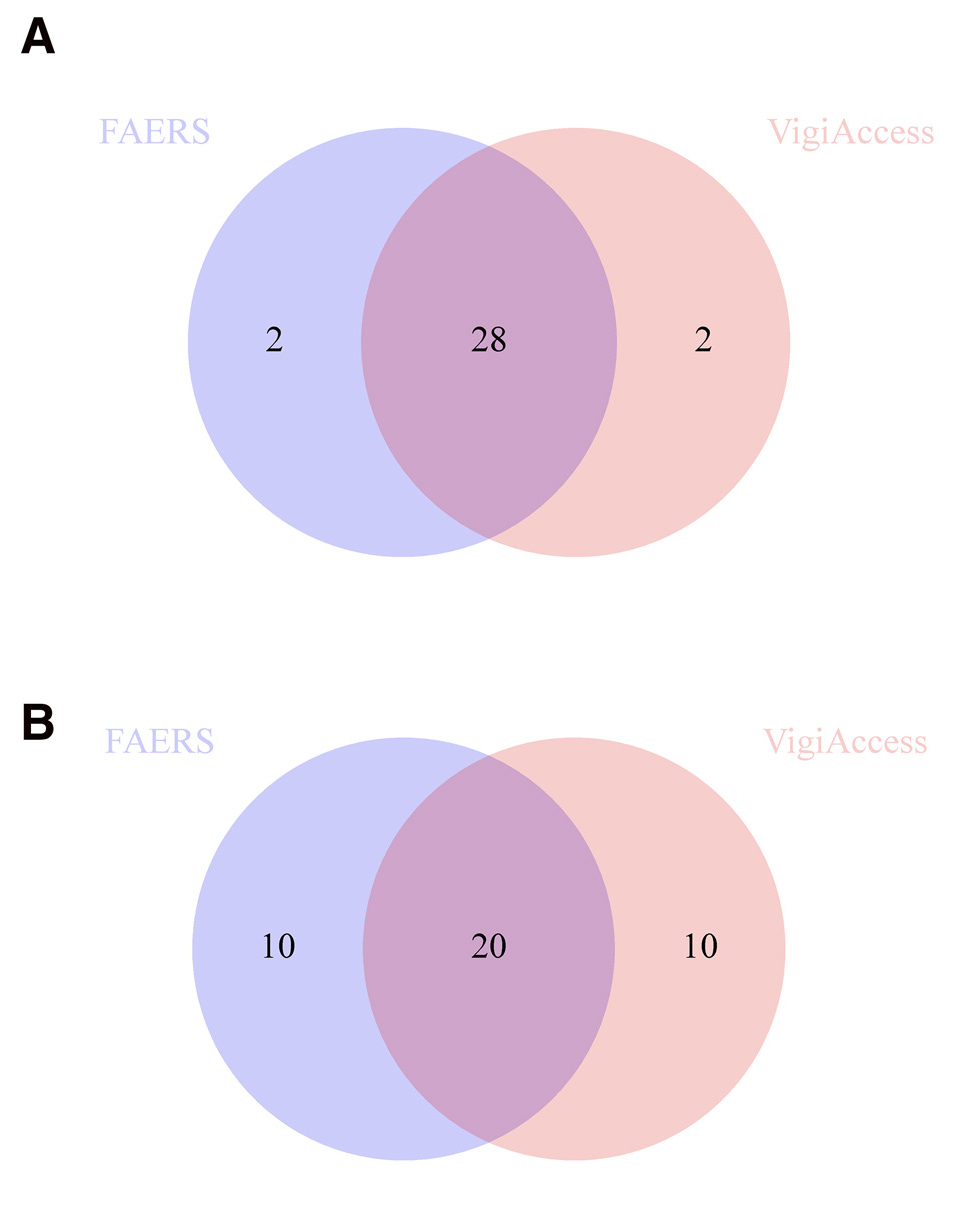
Venn diagrams of the top 30 preferred terms (PTs) for Zuranolone (A) and Brexanolone (B) by reported case number in two databases. FAERS = FDA adverse event reporting system.

In the present study, the PTs associated with AEs reported for the two drugs were analyzed, with a focus on AEs with high reporting frequencies. For Zuranolone, the reported AEs involved 281 PTs in FAERS (total case number: 1666) and 350 PTs in VigiAccess (total case number: 1629), respectively. Among the top 30 PTs ranked by the number of reported cases, 28 were consistent between the two databases (Fig. 3A). Specifically, the top 5 PTs for Zuranolone in FAERS were somnolence (n = 188, 11.28%), dizziness (n = 134, 8.04%), fatigue (n = 100, 6.00%), sedation (n = 51, 3.06%), and suicidal ideation (n = 51, 3.06%). In VigiAccess, the top 5 PTs for Zuranolone were identical to those in FAERS: somnolence (n = 258, 15.84%), dizziness (n = 178, 10.93%), fatigue (n = 144, 8.84%), sedation (n = 66, 4.05%), and suicidal ideation (n = 61, 3.74%). Detailed information on the top 30 PTs for Zuranolone is provided in Table 5.

**Table 5:** Top 30 PTs by number of reported cases of Zuranolone in two databases.

	FAERS			VigiAccess		
	PT	Case number	Percentage (%)	PT	Case number	Percentage (%)
1	Somnolence	188	11.28	Somnolence	258	15.84
2	Dizziness	134	8.04	Dizziness	178	10.93
3	Fatigue	100	6.00	Fatigue	144	8.84
4	Sedation	51	3.06	Sedation	66	4.05
5	Suicidal ideation	51	3.06	Suicidal ideation	61	3.74
6	Anxiety	48	2.88	Tremor	58	3.56
7	Tremor	42	2.52	Feeling abnormal	58	3.56
8	Feeling abnormal	36	2.16	Anxiety	58	3.56
9	Depression	34	2.04	Depression	46	2.82
10	Drug ineffective	34	2.04	Nausea	41	2.52
11	Memory impairment	26	1.56	Drug ineffective	36	2.21
12	Nausea	26	1.56	Gait disturbance	35	2.15
13	Gait disturbance	25	1.50	Memory impairment	29	1.78
14	Insomnia	20	1.20	Feeling drunk	29	1.78
15	Muscle twitching	19	1.14	Insomnia	27	1.66
16	Perinatal depression	19	1.14	Brain fog	27	1.66
17	Confusional state	18	1.08	Confusional state	26	1.60
18	Balance disorder	17	1.02	Balance disorder	24	1.47
19	Feeling drunk	17	1.02	Perinatal depression	23	1.41
20	Headache	15	0.90	Headache	23	1.41
21	Diarrhoea	14	0.84	Muscle twitching	22	1.35
22	Panic attack	14	0.84	Therapy cessation	18	1.10
23	Migraine	13	0.78	Panic attack	18	1.10
24	Anger	12	0.72	Diarrhoea	18	1.10
25	Therapy cessation	12	0.72	Hypoaesthesia	17	1.04
26	Depressed mood	11	0.66	Anger	17	1.04
27	Dysarthria	11	0.66	Dysarthria	16	0.98
28	Hypersomnia	11	0.66	Muscular weakness	15	0.92
29	Hypoaesthesia	11	0.66	Hypersomnia	15	0.92
30	Muscular weakness	11	0.66	Exposure via breast milk	15	0.92

Gray shading indicates PT shared by FAERS and VigiAccess; unshaded entries are database-specific PT.

FAERS = FDA adverse event reporting system, PT = preferred term.

For Brexanolone, the reported AEs involved 85 PTs in FAERS (total case number: 194) and 178 PTs in VigiAccess (total case number: 382), respectively. Among the top 30 PTs, 20 were overlapping between the two databases (Fig. 3B). The top 5 PTs for Brexanolone in FAERS were drug monitoring procedure incorrectly performed (n = 18, 9.28%), off-label use (n = 13, 6.70%), incorrect product administration duration (n = 13, 5.67%), anxiety (n = 9, 4.64%), and drug ineffective (n = 9, 4.64%). In contrast, the top 5 PTs in VigiAccess were product administration error (n = 32, 8.38%), anxiety (n = 27, 7.07%), off-label use (n = 24, 6.28%), drug ineffective (n = 18, 4.71%), and dizziness (n = 16, 4.19%). Details of the top 30 PTs for Brexanolone are presented in Table 6.

**Table 6:** Top 30 PTs by number of reported cases of Brexanolone in two databases.

	FAERS			VigiAccess		
	PT	Case number	Percentage (%)	PT	Case number	Percentage (%)
1	Drug monitoring procedure incorrectly performed	18	9.28	Product administration error	32	8.38
2	Off-label use	13	6.70	Anxiety	27	7.07
3	Incorrect product administration duration	11	5.67	Off-label use	24	6.28
4	Anxiety	9	4.64	Drug ineffective	18	4.71
5	Drug ineffective	9	4.64	Dizziness	16	4.19
6	Feeling abnormal	6	3.09	Fatigue	15	3.93
7	Exposure via breast milk	5	2.58	Drug monitoring procedure incorrectly performed	14	3.66
8	Headache	5	2.58	Product administration interrupted	14	3.66
9	Condition aggravated	4	2.06	Perinatal depression	14	3.66
10	Product administration interrupted	4	2.06	Feeling abnormal	12	3.14
11	Product use in unapproved indication	4	2.06	Therapeutic product effect incomplete	12	3.14
12	Depression	3	1.55	Incorrect product administration duration	11	2.88
13	Dizziness	3	1.55	Headache	11	2.88
14	Fatigue	3	1.55	Crying	11	2.88
15	Intrusive thoughts	3	1.55	Product use in unapproved indication	10	2.62
16	No adverse event	3	1.55	Medication error	9	2.36
17	Panic attack	3	1.55	Somnolence	9	2.36
18	Therapy cessation	3	1.55	Depression	9	2.36
19	Circumstance or information capable of leading to medication error	2	1.03	Suicidal ideation	9	2.36
20	Confusional state	2	1.03	Product use issue	8	2.09
21	Crying	2	1.03	Insomnia	7	1.83
22	Hypotension	2	1.03	Exposure via breast milk	6	1.57
23	Infusion site extravasation	2	1.03	Condition aggravated	6	1.57
24	Infusion site pain	2	1.03	Infusion site extravasation	6	1.57
25	Major depression	2	1.03	Infusion site pain	6	1.57
26	Menstruation irregular	2	1.03	Therapeutic product effect delayed	6	1.57
27	Myalgia	2	1.03	Sedation	5	1.31
28	Product administration error	2	1.03	Intrusive thoughts	5	1.31
29	Product use issue	2	1.03	Product preparation error	4	1.05
30	Pruritus	2	1.03	Sedation complication	4	1.05

Gray shading indicates PT shared by FAERS and VigiAccess; unshaded entries are database-specific PT.

FAERS = FDA adverse event reporting system, PT = preferred term.

### 3.4. Positive signals of Zuranolone and Brexanolone in FAERS database

Subsequently, the high signal strength of PTs associated with AEs reported for the two drugs was analyzed. Using the ROR and PRR methods for screening, a total of 78 positive signals (involving 9 SOCs) of Zuranolone were identified in the FAERS database (Table 7). Among these, 28 signals have not yet been included in the prescribing information. The top 5 PTs ranked by signal strength were perinatal depression (n = 19, ROR = 1284.87), feeling drunk (n = 17, ROR = 129.81), intrusive thoughts (n = 4, ROR = 83.18), sedation (n = 51, ROR = 72.68), and bradyphrenia (n = 10, ROR = 56.45).

**Table 7:** Positive signals at the PTs level for Zuranolone in FAERS database.

SOC	PTs	N	PRR (Chi_squared)	ROR (95% two-sided CI)
Nervous system disorders	Somnolence	188	37.79 (6727.61)	53.40 (45.01–63.34)
	Dizziness	134	11.59 (1308.08)	14.44 (11.93–17.48)
	Memory impairment	26	7.13 (131.73)	7.40 (5.00–10.96)
	Balance disorder*	17	9.18 (116.07)	9.40 (5.81–15.23)
	Migraine*	13	5.05 (38.38)	5.13 (2.96–8.89)
	Dysarthria	11	13.99 (120.02)	14.22 (7.83–25.82)
	Hypersomnia	11	15.95 (139.55)	16.22 (8.93–29.45)
	Hypoaesthesia*	11	3.37 (16.14)	3.41 (1.88–6.20)
	Motor dysfunction*	10	43.78 (375.03)	44.47 (23.78–83.15)
	Lethargy	9	7.58 (45.12)	7.68 (3.98–14.83)
	Amnesia*	8	6.07 (29.00)	6.13 (3.05–12.31)
	Cognitive disorder*	7	5.98 (24.28)	6.03 (2.86–12.71)
	Disturbance in attention	7	6.10 (24.98)	6.16 (2.92–12.97)
	Depressed level of consciousness*	6	5.95 (20.01)	5.99 (2.68–13.40)
	Dyskinesia*	5	5.95 (15.97)	5.99 (2.49–14.45)
	Speech disorder*	5	4.19 (9.19)	4.22 (1.75–10.18)
	Mental impairment*	4	6.71 (14.14)	6.74 (2.52–18.03)
	Aphasia*	3	4.58 (5.20)	4.60 (1.48–14.30)
	Coordination abnormal	3	15.79 (28.06)	15.86 (5.10–49.35)
	Incoherent	3	28.85 (55.08)	28.98 (9.31–90.24)
	Restless legs syndrome*	3	7.54 (11.11)	7.58 (2.44–23.56)
	Sensory disturbance*	3	11.53 (19.27)	11.58 (3.72–36.02)
Psychiatric disorders	Fatigue	100	4.80 (307.16)	5.52 (4.46–6.83)
	Sedation	51	66.89 (3229.88)	72.68 (54.55–96.84)
	Suicidal ideation	51	24.44 (1124.63)	26.50 (19.90–35.30)
	Anxiety	48	6.65 (227.06)	7.11 (5.30–9.54)
	Tremor	42	11.91 (410.89)	12.69 (9.28–17.36)
	Depression	34	7.14 (174.627)	7.49 (5.30–10.58)
	Insomnia*	20	3.47 (33.02)	3.55 (2.27–5.54)
	Perinatal depression	19	1246.22 (20250.23)	1284.87 (795.35–2075.69)
	Confusional state*	18	5.14 (56.27)	5.26 (3.29–8.41)
	Panic attack*	14	17.45 (200.87)	17.82 (10.49–30.28)
	Anger*	12	17.90 (174.88)	18.23 (10.29–32.29)
	Depressed mood	11	8.03 (60.95)	8.16 (4.49–14.81)
	Bradyphrenia*	10	55.57 (480.54)	56.45 (30.18–105.59)
	Mania*	9	26.67 (197.08)	27.04 (13.99–52.25)
	Irritability*	8	7.24 (37.02)	7.32 (3.64–14.70)
	Mood swings*	8	13.62 (81.36)	13.79 (6.86–27.70)
	Emotional disorder*	7	8.96 (41.86)	9.05 (4.29–15.06)
	Disorientation*	5	6.87 (19.56)	6.92 (2.87–16.68)
	Dissociation*	5	16.32 (57.37)	16.45 (6.82–39.68)
	Fear*	5	12.93 (43.72)	13.02 (5.40–31.41)
	Nervousness*	5	5.09 (12.60)	5.12 (2.12–12.35)
	Psychotic disorder*	5	7.78 (23.15)	7.83 (3.25–18.89)
	Restlessness*	5	6.36 (17.57)	6.41 (2.66–15.45)
	abnormal dreams	4	10.16 (24.51)	10.22 (3.82–27.33)
	Decreased interest*	4	35.25 (100.76)	35.46 (13.25–94.93)
	Depressive symptom	4	38.85 (111.73)	39.09 (14.60–104.66)
	Euphoric mood	4	18.43 (49.60)	18.54 (6.93–49.59)
	Initial insomnia*	4	16.24 (42.93)	16.34 (6.11–43.69)
	Intrusive thoughts*	4	82.66 (244.46)	83.18 (31.01–223.08)
	Middle insomnia*	4	8.82 (20.47)	8.87 (3.32–23.72)
	Mood altered*	4	6.48 (13.46)	6.51 (2.44–17.41)
	Nightmare	4	5.62 (10.92)	5.65 (2.11–15.10)
	Panic reaction*	4	25.24 (70.33)	25.40 (9.49–67.96)
	Dysphemia	3	29.73 (56.91)	29.87 (9.59–93.00)
	Paranoia*	3	8.79 (13.65)	8.83 (2.84–27.45)
	Sopor	3	6.73 (9.47)	6.76 (2.17–21.01)
	Tachyphrenia*	3	43.46 (85.29)	43.66 (14.01–136.02)
General disorders and administration site conditions	Feeling abnormal*	36	6.34 (157.97)	6.66 (4.76–9.33)
	Gait disturbance*	25	5.70 (92.93)	5.90 (3.95–8.80)
	Feeling drunk*	17	126.34 (1969.50)	129.81 (79.97–210.71)
	Gait inability*	10	7.63 (51.24)	7.74 (4.14–14.45)
	Crying*	7	10.89 (53.38)	11.00 (5.22–23.18)
	Feeling jittery*	6	16.57 (72.83)	16.72 (7.48–37.38)
	Hangover*	3	40.49 (79.16)	40.68 (13.06–126.72)
Musculoskeletal and connective tissue disorders	Muscle twitching	19	39.83 (679.05)	41.03 (25.97–64.82)
	Muscular weakness*	11	4.65 (28.10)	4.72 (2.60–8.57)
	Muscle spasms*	9	2.26 (5.13)	2.27 (1.18–4.39)
Surgical and medical procedures	Therapy cessation*	12	5.86 (43.71)	5.95 (3.36–10.54)
Social circumstances	Loss of personal independence in daily activities*	6	3.01 (6.21)	3.03 (1.36–6.78)
Ear and labyrinth disorders	Vertigo*	10	7.65 (51.40)	7.76 (4.15–14.49)
Eye disorders	Vision blurred*	10	3.06 (11.94)	3.09 (1.66–5.78)
	Diplopia	4	7.17 (15.51)	7.21 (2.70–19.26)
Injury, poisoning and procedural complications	Exposure via breast milk*	6	28.56 (132.90)	28.82 (12.89–64.46)
	Underdose	5	2.86 (4.33)	2.87 (1.19–6.92)
	Joint injury*	3	5.92 (7.85)	5.95 (1.91–18.49)
	Prescribed underdose	3	4.38 (4.80)	4.39 (1.41–13.66)

* Unlisted in the instructions.

CI = confidence interval, PRR = proportional reporting ratio, PT = preferred term, ROR = reporting odds ratio, SOC = system organ class.

For Brexanolone, screening via the same ROR and PRR methods yielded 10 positive signals (involving 4 SOCs) in the FAERS database (Table 8). The top 5 PTs by signal strength were drug monitoring procedure incorrectly performed (n = 18, ROR = 5500.65), intrusive thoughts (n = 3, ROR = 394.42), exposure via breast milk (n = 5, ROR = 154.70), incorrect product administration duration (n = 11, ROR = 73.17), and product administration interrupted (n = 4, ROR = 60.99).

**Table 8:** Positive signals at the PTs level for Brexanolone in FAERS database.

SOC	PT	N	PRR (χ^2^)	ROR (95% two-sided CI)
Injury, poisoning and procedural complications	Drug monitoring procedure incorrectly performed	18	4530.12 (72496.38)	5500.65 (3263.54–9271.28)
	Off-label use	13	2.37 (9.49)	2.57 (1.44–4.60)
	Incorrect product administration duration	11	65.38 (634.96)	73.17 (39.13–136.82)
	Exposure via breast milk	5	147.17 (586.07)	154.70 (62.92–380.40)
	Product administration interrupted	4	58.64 (172.63)	60.99 (22.43–165.83)
Psychiatric disorders	Anxiety	9	7.71 (46.55)	8.35 (4.21–16.56)
	Intrusive thoughts	3	382.85 (788.48)	394.42 (124.69–1247.61)
	Panic attack	3	23.10 (43.30)	23.77 (7.54–74.98)
General disorders and administration site conditions	Feeling abnormal	6	6.54 (23.07)	6.88 (3.02–15.70)
Surgical and medical procedures	Therapy cessation	3	9.05 (14.24)	9.30 (2.95–29.33)

CI = confidence interval, PRR = proportional reporting ratio, PT = preferred term, ROR = reporting odds ratio, SOC = system organ class.

### 3.5. Co-involving SOCs for Zuranolone and Brexanolone by positive AE signals at the PT level in FAERS

Through data mining, we found large differences in the number and intensity of AEs between Zuranolone and Brexanolone at the PT level. Based on the ROR method, SOCs commonly implicated in the AEs positive signal for two drugs include Injury, poisoning and procedural complications, Psychiatric disorders, General disorders and administration site conditions, Surgical and medical procedures. For Brexanolone, in the Psychiatric disorders, AEs only include Anxiety, Intrusive thoughts, and Panic attack. As for Zuranolone, some AEs, such as fatigue, sedation, suicidal ideation, tremor and depression were more common after Zuranolone administration.

**Figure 4.**
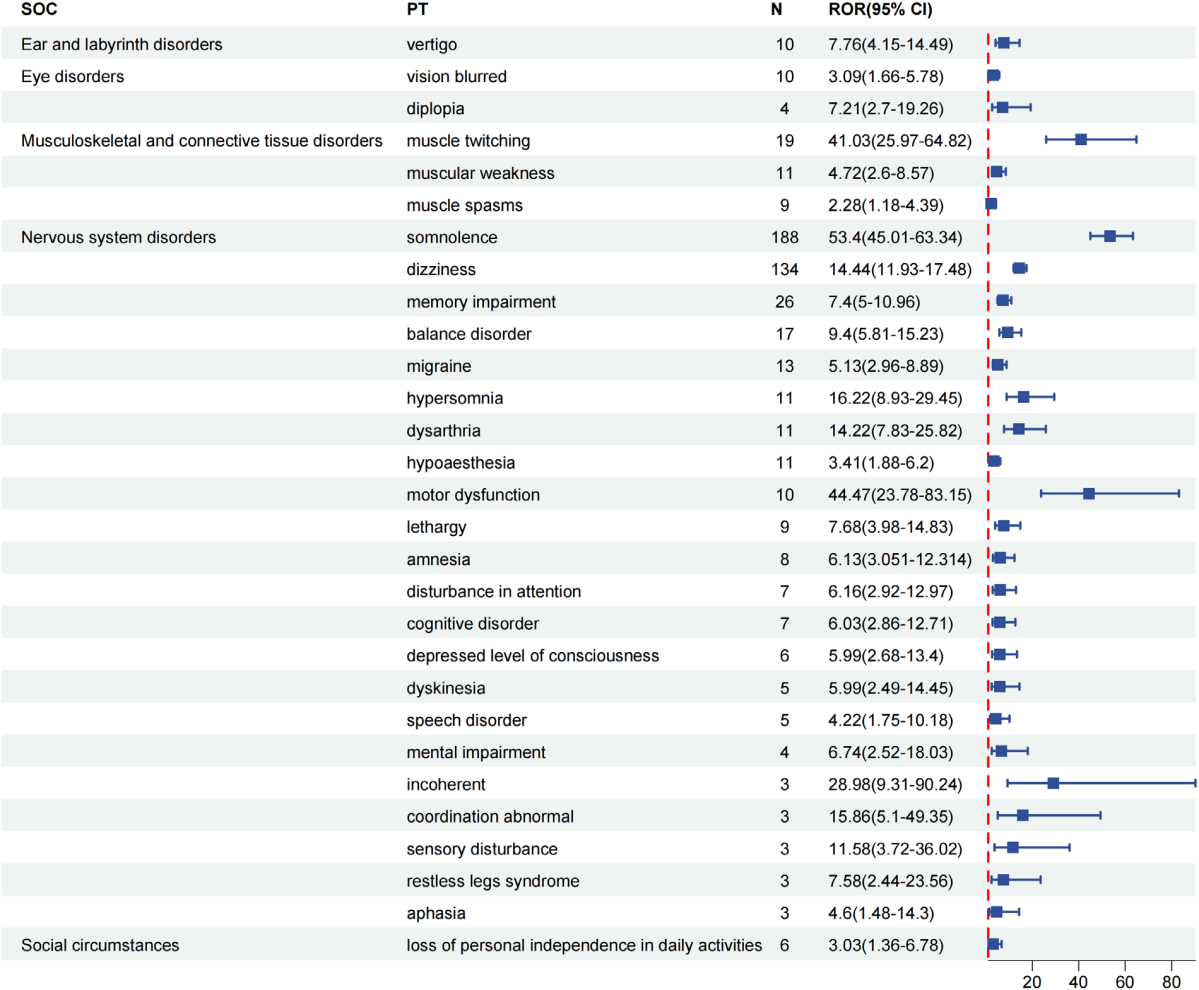
AE positive signals within SOC are individually accumulated by Zuranolone. CI = confidence interval, PT = preferred term, ROR = reporting odds ratio, SOC = system organ class.

Subsequently, we performed a detailed analysis of the AEs specifically associated with the SOCs of Zuranolone. Figure 4 lists AEs positive signals at the PTs level that were present in the SOCs that were individually accumulated by Zuranolone. Nervous system disorders and Musculoskeletal and connective tissue disorders with Zuranolone were found to be cause for alarm in this study.

## 4. Discussion

This real-world pharmacovigilance study, leveraging FAERS and VigiAccess, provides a systematic, comparative assessment of the post-marketing safety profiles of Zuranolone and Brexanolone. The clinical characteristics and SOC distributions of AEs for both drugs showed similar trends in FAERS and VigiAccess, which can act as auxiliary supporting evidence. In the two databases, Zuranolone had more AE reports than Brexanolone, possibly due to its better patient compliance as an oral drug and the resulting larger number of users. Both drugs are mainly used in females (except for those with unregistered gender), but their use in males may be off-label, consistent with other literature reports.^[24,25]^ Zuranolone’s top three AEs involved Nervous system disorders, General disorders and administration site conditions, Psychiatric disorders; and Brexanolone’s involved Injury, poisoning and procedural complications, General disorders and administration site conditions, Psychiatric disorders. Brexanolone, with Injury, poisoning and procedural complications reporting the highest number, is closely related to the drug’s specific delivery form. Zuranolone had one fatal serious outcome, whereas no death was documented for Brexanolone, which is consistent with previous findings.^[15,25,26]^ Stratified analysis of serious outcomes identified multiple suicidality-related events including completed suicide and suicidal ideation in Zuranolone reports, whereas no such AEs were recorded for Brexanolone. This disparity may result from selective reporting bias as well as intergroup imbalance in baseline characteristics and concomitant medications. Some Zuranolone patients with serious outcomes were co-administered SSRIs, serotonin-norepinephrine reuptake inhibitors, bupropion and other psychotropic agents, while Brexanolone recipients only received sedative anxiolytics. Incomplete clinical data in the FAERS database makes it impossible to rule out confounding effects from multiple medications and preexisting mental illness. Given the short post-marketing period and limited real-world evidence for two drugs, these safety signals should be interpreted cautiously.

This analysis detected some serious AEs, including death, hospitalization, life-threatening conditions, and other medically important events. Disproportionate analyses using PRR and ROR further yielded positive safety signals such as suicidal ideation, sedation, motor dysfunction, and loss of personal independence. Previous controlled trials have shown that both Zuranolone and Brexanolone achieve higher response and remission rates in patients with PPD, with mostly mild AEs,^[15,27,28]^ but this study confirmed the existence of serious AEs in real-world settings. The apparent discrepancy in AE severity between clinical trials and this study can be explained by several factors. Spontaneous reporting systems tend to overreport serious outcomes. In contrast to the highly selected trial populations, real-world patients have complex underlying illnesses and take multiple concomitant medicines. Additional contributors include post-marketing reporting bias and differences in dosing scenarios. The prominent severe AE signals identified in this study are similar to findings from prior FAERS studies,^[25,26]^ highlighting the necessity of monitoring these potential safety concerns. Many positive signals centered on Psychiatric Disorders, including perinatal depression, depression, suicidal ideation, anxiety, and panic attack. It should be noted that these manifestations are core clinical symptoms of PPD. Based on limited information from spontaneous reporting databases, we cannot distinguish whether these reported events are drug-related AEs, natural exacerbation of underlying mental illness, or clinical outcomes after treatment failure. Therefore, these positive signals contain obvious confounding interference from baseline disease. When assessing the safety of Zuranolone and Brexanolone, readers should not automatically classify these psychiatric AE reports as drug-induced AEs.

For the unique positive signals of Zuranolone at the SOC levels, such as Nervous system disorders, Eye disorders, and Ear and labyrinth disorders, their clinical significance lies in providing targeted monitoring directions for its safe use. This study found that Zuranolone has positive signals related to visual impairment (such as blurred vision, dizziness, etc), which are consistent with Zuranolone’ AEs not included in the package insert reported in the study by Chen et al.^[26]^ It suggests that clinicians need to pay attention to changes in patients’ visual function after medication, especially avoiding driving or operating heavy machinery within 12 hours after medication, which echoes the prompt regarding the impact on mobility in Zuranolone’s black box warning.^[29]^ For Brexanolone, attention should be paid to Psychiatric disorders, which is consistent with the research report by Jiang et al and the drug package insert.^[25]^ Owing to its low oral bioavailability, Brexanolone required a 60-hour continuous intravenous infusion and must be administered in a health care facility, as patients using this drug were at risk of excessive sedation or sudden loss of consciousness.^[30]^ Consequently, Brexanolone was only administered to patients through a restricted program known as the ZULRESSO Risk Evaluation and Mitigation Strategy.^[31,32]^ The AEs of Brexanolone also extensively involve SOCs such as General disorders and administration site conditions, Injury, poisoning and procedural complications, which are directly related to its intravenous infusion administration route. Such divergent SOC distributions of two drugs stem mainly from differences in launch year, administration route, target users and reporting rules, rather than indicating wider intrinsic safety risks for Zuranolone. In addition, considering that most patients with PPD are in the lactation period, this study found that the AEs of both drugs include exposure via breast milk. We further investigated the safety risks of breast milk exposure to the two agents. Disproportionality analysis revealed a positive signal only for exposure via breast milk. No statistically significant signals were detected for other maternal lactation-related AEs (lactation disorder, galactostasis, breast milk discoloration, one case each) or neonatal AEs (neonatal disorder, somnolence neonatal, rash neonatal, one case each). Among 6 Exposure via breast milk reports for Zuranolone, three were accompanied by neonatal AEs. Since each AE was only reported once, we can only conclude a potential neonatal safety risk. The other three records merely described maternal medication without neonatal AEs. No neonatal AEs were documented in the five Exposure via breast milk reports of Brexanolone. Safety data of these two drugs in breastfeeding populations remain sparse. These limited positive signals can inform clinical risk–benefit decisions.

Both Zuranolone and Brexanolone are neuroactive steroid agents indicated for the PPD. As the PAMs of GABA_A_ receptors, they modulate both synaptic and extrasynaptic GABAA receptors, thereby upregulating GABA_A_ receptors expression and enhancing GABAA signaling.^[33,34]^ The AEs in the CNS reported with Zuranolone, including somnolence, memory impairment, motor dysfunction, and sensory disturbance, as well as Psychiatric Disorders observed with both Zuranolone and Brexanolone such as anxiety, intrusive thoughts, and panic attack, may be closely associated with their broad modulation of GABA_A_ receptors and subsequent imbalance in neurotransmitter networks. GABA_A_ receptors represent the primary inhibitory neurotransmitter receptors in the CNS, which are mainly composed of two α subunits (α1–α6), two β subunits (β1–β3), and one additional subunit (γ1–γ3, δ, ε, π, or θ), with widespread distribution throughout the brain.^[35]^ Among these subunits, the α subtype plays a particularly critical role in determining the functional properties of GABA_A_ receptors. Notably, Brexanolone and Zuranolone lack strict subtype selectivity and can act on receptors containing multiple α subunits, including α1, α2, α3, and α5.^[36]^ Specifically, the α1 subunit is involved in sedation and hypnosis, α2 and α3 subunits are linked to anxiety and depression, and the α5 subunit contributes to cognitive regulation. Excessive activation of the α1 subunit by these agents frequently induces sedation, drowsiness, and dizziness, while modulation of the α5 subunit may trigger transient cognitive deficits.^[26]^ As PAMs, Brexanolone and Zuranolone potentiate GABA binding, prolong the opening of chloride channels, and strengthen the inhibitory effects of GABAergic neurotransmission. Such excessive inhibition can spread across multiple brain regions including the cerebral cortex, hippocampus, and thalamus, reducing overall brain excitability and leading to depressed consciousness and excessive sedation.^[37]^ Overactivation of the GABAergic system may also indirectly disrupt excitatory neurotransmitter systems including serotonin, dopamine, and norepinephrine. Enhanced GABAergic inhibition can suppress the firing of serotonergic neurons in the dorsal raphe nucleus, reducing serotonin release and contributing to psychiatric symptoms such as panic attack, anxiety and depression.^[38,39]^ Meanwhile, inhibition of dopaminergic neurons in the nigrostriatal pathway may lead to mild motor incoordination, presenting as dizziness and unsteady gait. Regarding the SOC-specific positive signals unique to Zuranolone, such as Eye disorders and Ear and labyrinth disorders, these may arise from the drug’s impacts on the CNS and vestibular function. Specifically, vertigo is caused by vestibular compensation disorders, which induce both static and dynamic vestibular symptoms.^[40]^ The differences in the SOC levels of these two drugs may be attributed to the following factors: Zuranolone, a derivative obtained by modifying Brexanolone with the introduction of a pyrazole substituent in its molecular structure, has better pharmacokinetic properties; in addition, the two drugs lack strict subtype selectivity of the GABAA receptors, which is also an important reason for the differences in the SOC involved in their adverse reactions.

However, this study is still limited by the inherent shortcomings of spontaneous reporting databases. Firstly, reporting bias is unavoidable: severe and rare AEs are more likely to be reported in clinical practice, while mild and common AEs may be overlooked, leading to overestimation or underestimation of some signals. Secondly, the databases lack patients’ complete baseline information (such as age, comorbidities, and concurrent medications) and medication details (such as dosage and treatment course), making it impossible to rule out the impact of confounding factors on the occurrence of AEs and difficult to analyze the dose-response relationship. Additionally, reports in FAERS and VigiAccess are derived from the U. S., which may restrict the generalizability of the research conclusions to different populations. Finally, ROR and PRR quantitative signal screening was only feasible in FAERS. VigiAccess database merely supplies summarized SOC/PT reporting frequencies without complete individual case data, making equivalent disproportionality analysis impossible. Thus, VigiAccess results only serve as descriptive auxiliary evidence.

## 5. Conclusions

This pharmacovigilance study showed Zuranolone’s AEs mainly involved the Nervous system disorders and Psychiatric disorders, while Brexanolone’s primarily included Injury, poisoning and procedural complications, and Psychiatric disorders. Although Zuranolone provides a novel therapeutic option for PPD, this study clarifies the post-marketing safety profiles of both drugs. These findings enhance clinical awareness of Zuranolone-linked AE signals, help healthcare professionals mitigate AE risks, and provide real-world safety data for its rational use. As a retrospective analysis built on spontaneous reporting databases, this study has inherent limitations. Larger clinical cohorts, prescription exposure data and prospective trials are still needed to validate these observations.

## Acknowledgments

This research was supported by the Special Project on Maternal and Child Health Development in Haidian District Maternal and Child Health Hospital (HFKT2026-08). We sincerely thank FAERS and VigiAccess databases for providing critical pharmacovigilance data that supported this study.

## Author contributions

**Conceptualization:** Guanbo Xie, Jingxian Yu.

**Funding acquisition:** Guanbo Xie.

**Investigation:** Guanbo Xie, Huili Weng.

**Software:** Guanbo Xie.

**Formal analysis:** Huili Weng.

**Supervision:** Liqun Chi.

**Writing – original draft:** Guanbo Xie.

**Writing – review & editing:** Jingxian Yu, Liqun Chi.


