# Supplementary material for: Real-world adverse event profiles of Zuranolone and Brexanolone based on FAERS and VigiAccess databases: An observational study
Source: Medicine (Baltimore). 2026 Jul 17;105(29):e49845. doi: 10.1097/MD.0000000000049845 (PMC13384562; doi:10.1097/MD.0000000000049845)
Supplement: Supplementary file 1 [file medi-105-e49845-s001.docx]

**Table S1.** PT composition of serious adverse outcome cases for Zuranolone and Brexanolone,

| Zuranolone: PT Composition of Serious Outcome Cases | | | | | |
| --- | --- | --- | --- | --- | --- |
| Seriou Outcome | PT | Case number (n) | Seriou Outcome | PT | Case number (n) |
| Death | Completed suicide | 1 | Other Serious (Important Medical Event) | Sedation | 2 |
|  | Perinatal depression | 1 |  | Feeling of despair | 2 |
| Hospitalization - Initial or Prolonged | Dizziness | 5 |  | Nausea | 2 |
|  | Anxiety | 2 |  | Off label use | 2 |
|  | Mental disorder | 2 |  | Feeling abnormal | 2 |
|  | Suicidal ideation | 2 |  | Fall | 2 |
|  | Muscle twitching | 2 |  | Feeling drunk | 2 |
|  | Heart rate decreased | 2 |  | Insomnia | 2 |
|  | Drug ineffective | 2 |  | Panic attack | 2 |
|  | Gait disturbance | 2 |  | Initial insomnia | 2 |
|  | Depressed mood | 1 |  | Hypomania | 1 |
|  | Intestinal obstruction | 1 |  | Influenza like illness | 1 |
|  | Somnolence | 1 |  | Acute kidney injury | 1 |
|  | Grief reaction | 1 |  | Product dose omission issue | 1 |
|  | Muscular weakness | 1 |  | Visual | 1 |
|  | Insomnia | 1 |  | Intentional self-injury | 1 |
|  | Intentional overdose | 1 |  | Post-traumatic stress disorder | 1 |
|  | Suicide attempt | 1 |  | Balance disorder | 1 |
|  | Blood pressure fluctuation | 1 |  | Malaise | 1 |
|  | Syncope | 1 |  | Abortion spontaneous | 1 |
|  | Panic attack | 1 |  | Maternal exposure during pregnancy | 1 |
|  | Confusional state | 1 |  | Nightmare | 1 |
|  | Dysarthria | 1 |  | Tic | 1 |
|  | Transient ischaemic attack | 1 |  | Convulsive threshold lowered | 1 |
|  | Neurological symptom | 1 |  | Illicit prescription attainment | 1 |
|  | Aphasia | 1 |  | Hot flush | 1 |
|  | Disturbance in attention | 1 |  | Pain | 1 |
|  | Eyelid function disorder | 1 |  | Visual impairment | 1 |
|  | Fatigue | 1 |  | Crying | 1 |
|  | Memory impairment | 1 |  | Feeling guilty | 1 |
|  | Mental impairment | 1 |  | Abnormal behaviour | 1 |
|  | Nausea | 1 |  | Decreased interest | 1 |
|  | Vertebrobasilar artery dissection | 1 |  | Homicidal ideation | 1 |
|  | Behaviour disorder | 1 |  | Auditory | 1 |
|  | Major depression | 1 |  | Ankle fracture | 1 |
|  | Perinatal depression | 1 |  | Syncope | 1 |
|  | Therapeutic product effect decreased | 1 |  | Loss of personal independence in daily activities | 1 |
|  | Therapeutic response shortened | 1 |  | Transient ischaemic attack | 1 |
|  | Blood pressure increased | 1 |  | Diarrhoea | 1 |
|  | Hypotension | 1 |  | Headache | 1 |
| Life-Threatening | Suicidal ideation | 2 |  | Self-injurious ideation | 1 |
|  | Condition aggravated | 1 |  | Tremor | 1 |
|  | Panic attack | 1 |  | Hypophagia | 1 |
|  | Sedation | 1 |  | Brain fog | 1 |
|  | Akathisia | 1 |  | Vision blurred | 1 |
| Other Serious (Important Medical Event) | Suicidal ideation | 41 |  | Weight increased | 1 |
|  | Depression | 6 |  | Tearfulness | 1 |
|  | Anxiety | 5 |  | Feeding disorder | 1 |
|  | Perinatal depression | 4 |  | Tachyphrenia | 1 |
|  | Fatigue | 4 |  | Substance abuse | 1 |
|  | Somnolence | 4 | Required Intervention to Prevent Permanent Impairment/Damage | Somnolence | 2 |
|  | Psychotic disorder | 4 |  | Therapy cessation | 2 |
|  | Memory impairment | 4 |  | Anxiety | 1 |
|  | Dizziness | 4 |  | Fatigue | 1 |
|  | Seizure | 3 |  | Feeling abnormal | 1 |
|  | Intrusive thoughts | 3 |  | Hypoaesthesia | 1 |
|  | Hallucination | 3 |  | Loss of personal independence in daily activities | 1 |
|  | Mania | 3 |  | Mood swings | 1 |
|  | Dissociation | 3 |  | Affective disorder | 1 |
|  | Depressed mood | 3 |  | Dizziness | 1 |
|  | Drug ineffective | 3 |  | Fall | 1 |
|  | Paralysis | 2 |  | Joint injury | 1 |
| Brexanolone: PT Composition of Serious Outcome Cases | | | | | |
| Seriou Outcome | PT | Case number (n) | Seriou Outcome | PT | Case number (n) |
| Hospitalization - Initial or Prolonged | Brief psychotic disorder | 1 | Other Serious (Important Medical Event) | Sedation complication | 1 |
|  | Confusional state | 1 |  |  |  |
|  | Extrapyramidal disorder | 1 |  | Electrocardiogram qt prolonged | 1 |
|  | Incorrect product administration duration | 1 |  |  |  |
|  | Product preparation error | 1 |  | Incorrect product administration duration | 1 |
|  | Thinking abnormal | 1 |  |  |  |
|  | With postpartum onset | 1 |  | Tachycardia | 1 |
